# Blocks of chromosomes identical by descent in a population: Models and predictions

**DOI:** 10.1371/journal.pone.0187416

**Published:** 2017-11-02

**Authors:** Mathieu Tiret, Frédéric Hospital

**Affiliations:** UMR 1313 Génétique Animale et Biologie Intégrative, INRA, Jouy-en-Josas, France; China University of Science and Technology, CHINA

## Abstract

With the highly dense genomic data available nowadays, ignoring linkage between genes would result in a huge loss of information. One way to prevent such a loss is to focus on the blocks of chromosomes shared identical by descent (IBD) in populations. The development of the theoretical framework modelling IBD processes is essential to support the advent of new tools such as haplotype phasing, imputation, inferring population structure and demographic history, mapping loci or detecting signatures of selection. This article aims to present the relevant models used in this context, and specify the underlying definitions of identity by descent that are yet to be gathered at one place. In light of this, we derived a general expression for the expected IBD block length, for any population model at any generation after founding.

## 1 Introduction

Two alleles are said to be identical by descent (IBD) if they are inherited copies of the same ancestral allele. In the past, IBD was mostly studied at one locus or a few independent loci. Nowadays, with the advent of Next Generation Sequencing techniques, new models and concepts integrating several loci at once (‘multilocus IBD’) have become prominent in genome scan analyses. The idea is to take full account not only of the high number of available marker loci, but also of their high density per genome length (in Morgan). In such analyses, linkage and linkage disequilibrium can no longer be ignored as was the case in the past with scarcer maps. Indeed, integrating haplotype information in genome scan analyses adds value to multilocus IBD studies [1]. In this paper, we will focus on IBD blocks of chromosomes, or contiguous IBD loci, and thereby account for linkage between loci. Note that it is also possible to study probabilities of several disruptive loci to be IBD [2, 3], but this is a different approach of multilocus IBD that will not be considered here.

Developing the theoretical framework underlying IBD processes has become essential for the development of new tools suitable for high density genomic data, such as haplotype phasing and imputation [4], inference of population structure and demographic history [4, 5], mapping loci or detecting signatures of selection [6, 7].

In the literature, several alternative definitions of an IBD block exist. We will first try to properly define the concepts and clarify implicit considerations for each definition. Then, we will present some of the relevant models used to study IBD blocks in a population. Practical applications of these models were thoroughly reviewed in Browning’s article [1].

### 1.1 Diversity of definitions

From here onwards, let us call a ‘locus’ a common position over a set of *n* homologous chromosomes, and a ‘segment’ a set of adjacent loci. The concept of IBD is always relative to a founder population. It could be defined for *k* loci over *n* homologous chromosomes. It has already been thoroughly defined at one locus (*k* = 1) for any number of homologous chromosomes, and we are trying here to define it properly for a segment, for any *k* and any *n*. Paraphrasing some articles of the literature on IBD studies [8–11], we suggest in this article that *n* homologous tracts of chromosomes are IBD if they are inherited copies of the same ancestral homologous tract of a chromosome. By the definition of segment, we are only considering homologous chromosomes, excluding transposable elements. Specifying that they are ‘inherited’ excludes horizontal gene transfer.

Identity by descent is a powerful concept with which it is possible to describe how genetic material is transmitted or lost over time. Assuming that genetic material could be split into a ‘container’ and a ‘content’, studying the containers independently of the content is a matter of IBD. On the other hand, studying the content is a matter of identity by state (IBS), not of IBD. Therefore, everything that concerns the content, namely the sequence, such as IBS or mutations, is not accounted for here: they are issues of allelic variation, not of descent. One should account for mutations only when approximating IBD through IBS. On the contrary, recombination events have to be taken fully into account. In this paper, we will not be considering crossovers among non-homologous chromosomes. There are two types of crossovers: those that occur between two tracts that are IBD and thus invisible; and the others that are called ‘junctions’ [12, 13]. Describing and predicting the dynamics of junctions is a core part of IBD studies.

Furthermore, we could distinguish two types of multilocus IBD, relaxed or strict. Relaxed IBD at a segment is a relation between *n* homologous chromosomes that are IBD at every locus of the segment, each locus being not necessarily of the same ancestral origin as its adjacent loci. Strict IBD requires that in addition the *n* homologous chromosomes have the same ancestral origin at each locus of the segment.

**Fig 1:**
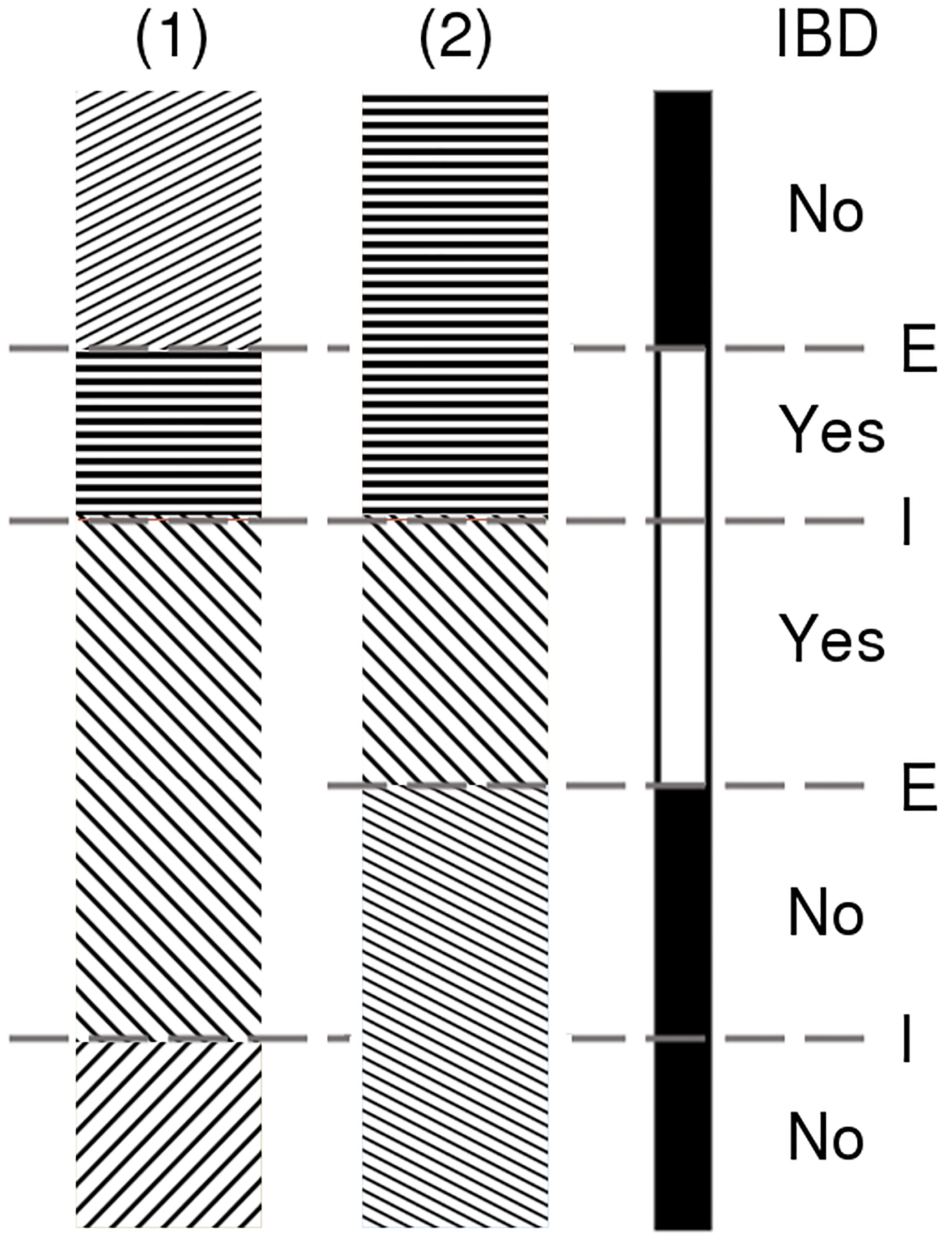
Two homologous chromosomes, labelled ‘(1)’ and ‘(2)’, some generations after founding. Different patterns on the chromosomes represent the different ancestral origins. The third axis, labelled ‘IBD’, is the IBD axis on which white parts indicate the IBD tracts, and black parts the non-IBD tracts. Each junction is projected on this axis and labelled ‘E’ if it is an external junction, and ‘I’ if it is an internal junction. When considering the relaxed IBD, there is only one IBD block, whereas when considering the strict IBD, there are two of them.

When considering *n* homologous chromosomes, one could project on an axis whether or not these chromosomes are IBD for each locus. This axis is here called the IBD axis (see Fig 1). On this axis, we could clearly distinguish IBD tracts and non-IBD tracts. A junction is external if its projection on this axis delimits an IBD and a non-IBD tract, and is internal if its projection is within an IBD or a non-IBD tract. We define a relaxed IBD block as a contiguous IBD tract delimited by external junctions or tips of chromosomes, without any external junction in it. In addition, strict IBD blocks are also delimited by internal junctions that are within IBD tracts. There is no junction in a strict IBD block. Depending on the definition, there could be a different number of IBD blocks, as can be seen in the example in Fig 1, on which there is either one relaxed IBD block or two strict IBD blocks. Hereafter, we only consider relaxed IBD.

### 1.2 Modelling choices

In the literature, only two values of *n* were studied, 2 and the population size *N* (or 2*N* for diploid populations), although intermediate values of *n* could be considered as well. When *n* = 2 in a diploid population, some models focus on pairs of homologous chromosomes within individuals, and IBD is then called ‘homozygosity by descent’ [14, 15], and some on random pairs of homologous chromosomes in a population [8].

For all the definitions presented above, the locations on the chromosome could either be modelled as continuous [8, 10, 15] or as discrete objects [16]. In fact, all these cited papers treat the underlying chromosome as a continuum, but some maybe model transitions in IBD state from (discrete) locus to locus—as is natural to do when dealing with actual data at marker loci (anonymous referee, personal communications).

Genome length is measured in Morgan and crossovers are usually supposed to follow the no-interference recombination model of Haldane [17]: at each meiosis, each chromosome of length *l* (in Morgan) undergoes crossovers, whose number follows a Poisson law of parameter *l* and whose positions are independent random variables each with a uniform distribution. Therefore, the crossover events follow a Poisson process of rate 1 in the Haldane recombination model. As long as the Haldane recombination model is valid, measuring the genome length in Morgans as in every article cited here, or studying the consequences of variation in recombination rate along the chromosome [18] are strictly equivalent.

One of the major problems of this field is a proper prediction of how IBD evolves over time in a population. There are several ways to quantify IBD in a population, the most important ones, considering *n* homologous chromosomes, being the number of IBD blocks, the length of one IBD block, and the total length of IBD blocks over these *n* homologous chromosomes. This paper extends previous studies on the evolution over time of the distributions of these quantities, or of their moments, in stochastic models of population genetics [5, 8, 12, 15, 19, 20]. The difficulty lies in the accumulation of junctions and the merging of IBD blocks over time. In the next section, we will review two major types of forward models, either based on random walks, or on renewal processes.

## 2 Models

### 2.1 Random walk on a hypercube

Considering IBD shared among *n* = 2*N* homologous chromosomes inherited from two different founder chromosomes only (denoted 0 and 1), it is possible to derive the true distribution of the relevant quantities of multilocus IBD as follows. One of the relevant quantities we will be focusing on is the total length of IBD blocks over the chromosome, or ‘total IBD length’. At each locus, one chromosome is denoted 0 or 1 depending on which founder it originates from. At each locus, the population of *n* homologous chromosomes is hence a *n*-tuple of 0’s and 1’s. Furthermore, we assume the continuous model of a chromosome, so that there is an infinite number of possible positions on a chromosome where a crossover could occur. Therefore, new crossover has a zero probability to occur in a location of another existing crossover. In a process whose states are the *n*-tuples of 0’s and 1’s and the time parameter is the map distance along the chromosomes, at most one coordinate of the *n*-tuple changes at each position, because of the continuous model. This process may thus be modelled as a realisation of a particular Markov process, namely a continuous-time Markov random walk on the vertices of a *n*-hypercube. Only two vertices of the hypercube are of interest, (0, …, 0) and (1, …, 1) which correspond to the states in which the population is IBD. The other vertices are the non-IBD states. Donnelly [21] first considered this problem and succeeded to reduce the dimension of the problem by gathering the vertices in what he called orbits, and provided the corresponding transition rate matrix.

Ball & Stefanov [10] used this theoretical framework to derive the exact characteristic function of the total IBD length among half-sibs, assuming that the number of non-IBD blocks was Poisson distributed. From this work, it is possible to deduce the exact probability of survival of the parental genetic material over one generation. Walters & Cannings [22] provided a general method for finding the density of the total IBD length, that could be applied to any unilineal relationship, and more specifically they provided the density of total IBD length for a grandparent-grandchild relationship.

Martin & Hospital [20] considered a particular lineage of recombinant inbred lines of 2 or 4 homologous chromosomes undergoing generations of respectively self-crossings or full-sib matings. In this model, each point of a chromosome is denoted 0 and 1 depending on which chromosome of the previous generation it inherited from [23]. Considering *g* generations, and modelling the chromosome as a continuum, the problem could also be modelled as a random walk on a *g*-hypercube. With this model, Martin & Hospital [20] studied the distribution of the length of IBD blocks depending on their positions on a semi-infinite chromosome, and showed that the successive blocks are almost independent and that the block at the origin of the chromosome was larger than the others. This counter-intuitive result is mainly due to the non-exponential distribution of block lengths (see Eq 5 and surrounding text in [20]), and could also be observed on finite length chromosomes (data not shown).

Using a random walk, it is possible to derive the distribution of IBD quantities, but only when assuming very particular pedigrees. In the next section, we will present theoretical results for a more general population model albeit only means have been accurately derived so far.

### 2.2 Renewal process in a random mating population

In this section, we will study the evolution over time of the relaxed IBD shared among pairs of homologous chromosomes (*n* = 2) in any kind of diploid population descending from a founder population. Without loss of generality, we will hereafter focus on pairs of homologous chromosomes within individuals, or homozygosity by descent, and provide an expression of the expected length of IBD blocks. The length and the number of IBD blocks per chromosome are not independent, and this dependency is very difficult to handle. Therefore, we have tried to develop a workaround by using quantities that are not affected by this dependency.

Let P denote the set of all possible populations of a stochastic or deterministic population model M. To any population p∈P, the model also assigns a probability P(p), which is the probability of encountering this population. One population is constituted of individuals, all carrying zero or more IBD blocks, so that a population is both a set of individuals and a set of IBD blocks. In other words, the model also assigns probabilities, indirectly though, to all possible individuals and all possible IBD blocks.

Let us now consider that every population p∈P has the same number *N* of individuals. For a given population *p*, the fact that an individual *i* is within this population is denoted *i* ∈ *I*_*p*_, and similarly, that an IBD block *b* is carried by an individual within this population is denoted *b* ∈ *B*_*p*_. For any individual *i* ∈ *I*_*p*_, we denote *d*_*i*_ its total IBD length and *k*_*i*_ the number of IBD blocks it carries. We also denote *m*_*p*_ = ∑_*i* ∈ *I*_*p*__ *k*_*i*_ the total number of IBD blocks in the population *p*. For any IBD block *b* ∈ *B*_*p*_, we denote *l*_*b*_ its length. Let *X* be an IBD block randomly drawn from $\cup_{p\in P}B_{p}$, and *L* its length. We are interested in deriving the expected length E(L) of a randomly drawn IBD block. If $P^{*}$ is the set of populations in which there is at least one IBD block, we have:

$$\begin{array}{ccc} \forall p\in P^{*},\;E\left(L|\;X\in B_{p}\right) & = & \frac{\sum_{b\in B_{p}}l_{b}}{m_{p}}\\ & = & \frac{\sum_{i\in I_{p}}d_{i}}{m_{p}} \end{array}$$ (1)

where *X* ∈ *B*_*p*_ means that the block *X* belongs to the population *p*. The population *p* was drawn through sampling a block, and is then size-biased: populations do not have the same number of IBD blocks, therefore sampling a block is not an unbiased way of drawing a population. One could then state that:

$$\begin{array}{c} \forall p\in P^{*},\;P\left(X\in B_{p}\right)=\frac{P\left(p\right)\cdot m_{p}}{\sum_{q\in P^{*}}P\left(q\right)\cdot m_{q}} \end{array}$$ (2)

where *q* is a population of $P^{*}$, and assuming that $P(X\in B_{p})$ is defined for the population model M, or equivalently that $\sum_{q\in P^{*}}P\left(q\right)\cdot m_{q}$ does not diverge towards infinity. *X* ∈ *B*_*p*_ indicates a unique population and the union of these populations, considering all the possible *X*, is $P^{*}$. Therefore, using Eqs (1) and (2), and the law of total expectation, one could derive that:

$$\begin{array}{ccc} E(L) & = & E_{P^{*}}\left(E\left(L|\;X\in B_{p}\right)\right)\\ & = & \sum_{p\in P^{*}}P\left(X\in B_{p}\right)\cdot E\left(L|\;X\in B_{p}\right)\\ & = & \sum_{p\in P^{*}}\frac{P\left(p\right)\cdot m_{p}}{\sum_{q\in P^{*}}P\left(q\right)\cdot m_{q}}\cdot \frac{\sum_{i\in I_{p}}d_{i}}{m_{p}}\\ & = & \frac{\sum_{p\in P^{*}}P\left(p\right)\cdot \sum_{i\in I_{p}}d_{i}}{\sum_{q\in P^{*}}P\left(q\right)\cdot \sum_{i\in I_{q}}k_{i}} \end{array}$$ (3)

In parallel, let *Y* be an individual randomly drawn from $\cup_{p\in P}I_{p}$, *D* its total IBD length and *K* the number of IBD blocks it carries. One could trivially state that:

$$\begin{array}{c} \forall p\in P,\;P\left(Y\in I_{p}\right)=P\left(p\right) \end{array}$$ (4)

where *Y* ∈ *I*_*p*_ means that the individual *Y* belongs to the population *p*. This population *p* was drawn through sampling an individual, therefore there is no size-bias, because all populations in P have the same number of individuals *N*. Also, one could derive that:

$$\begin{array}{c} E(D|Y\in I_{p})=\frac{\sum_{i\in I_{p}}d_{i}}{N} \end{array}$$ (5)

$$\begin{array}{c} E(K|Y\in I_{p})=\frac{\sum_{i\in I_{p}}k_{i}}{N} \end{array}$$ (6)

Finally, using all the above, one obtains:

$$\begin{array}{ccc} E(L) & = & \frac{\sum_{p\in P^{*}}P\left(Y\in I_{p}\right)\cdot \sum_{i\in I_{p}}d_{i}/N}{\sum_{q\in P^{*}}P\left(Y\in I_{q}\right)\cdot \sum_{i\in I_{q}}k_{i}/N}\\ & = & \frac{E(D)-\sum_{p\in P\setminus P^{*}}P\left(Y\in I_{p}\right)\cdot E\left(\;Y\in I_{p}\right)}{E\left(K\right)-\sum_{q\in P\setminus P^{*}}P\left(Y\in I_{q}\right)\cdot E\left(K|\;Y\in I_{q}\right)}\\ & = & \frac{E(D)}{E(K)} \end{array}$$ (7)

where $P\setminus P^{*}$ is the set of populations in which there are no IBD blocks, and knowing that *Y* ∈ *I*_*p*_ indicates a unique population and that the union of all these populations, considering all the possible *Y*, is P.

Eq (7), which is the key point of this article, is valid at any time *t* after founding, for any diploid population model and for any chromosome model (continuous or discrete). The only assumptions are that all populations have the same size at generation *t* and that $\sum_{q\in P^{*}}P\left(q\right)\cdot m_{q}$ does not diverge towards infinity. In other words, if the population size is only dependent on generation *t*, then Eq (7) is independent of any demographic structure of the population (subdivision in one or several demes, constant population size or not, panmictic or not…), and also of any evolutionary pressure (any kind of selection, any migration rate, recessive deleterious load…): it is up to E(D) and E(K) to handle these dependencies. Eq (7) could also be extended to any number *n* of homologous chromosomes, the only difference being that *Y* would be a randomly drawn *n*-tuple of homologous chromosomes. These *n* homologous chromosomes should however be all in the same population. Eq (7) is therefore of a very powerful and general use.

Let us now derive the expressions of E(D) and E(K) for some population models. Let E(H) and E(Z) be respectively the expected non-IBD proportion of a randomly drawn individual (ranging from 0 to 1) and the expected number of external junctions per Morgan within a randomly drawn individual.

In his seminal work, Stam [15] studied the relaxed IBD in a population and provided an approximation of E(H) and the exact value of E(Z). Stam’s E(Z) is so far the only quantity that successfully integrates the accumulation of junctions through time in a whole population. He considered a panmictic monoecious diploid population without selfing and undergoing drift only. The founder population was assumed to be entirely constituted of unrelated and non-inbred individuals (i.e. none of the chromosome pair was IBD). He modelled the chromosomes as continuous objects, and assumed the recombination model of Haldane.

In the second part of his article, Stam [15] found that the expected length L* of an IBD block would be expressed as follows:

$$\begin{array}{c} L^{*}=\frac{1-E(H)}{0.5\cdot E(Z)} \end{array}$$ (8)

assuming that IBD and non-IBD block lengths were exponentially distributed each with its own parameter. Chapman [8] extended Stam’s work and found the same result as Eq (8), without assuming exponential distributions of the block lengths. Stam [15] explicitly assumed stationarity of the IBD process. Though not explicitly assuming stationarity, Chapman [8] used equation (7.3) from Karlin’s book ([24]: p.199), which does assume stationarity of the IBD process. Both of these articles therefore assumed stationarity, implying that the processes ‘began indefinitely far in the past’ ([24]: p.199). The x-axis of processes described in Karlin’s book [24] was time, whereas the x-axis of processes studied here is the genetic map. So strictly speaking, assuming stationarity amounts to assuming that in Eq (8) the chromosome length was infinite.

If the chromosome length is assumed to be infinite, we get E(D)=1-E(H) and E(K)=0.5·E(Z), so that our Eq (7) is equivalent to Eq (8). If the chromosome is however of finite length *l*, we use the results from Fisher [12] to obtain the following:

$$\begin{array}{c} E(D)=l\cdot (1-E(H)) \end{array}$$ (9)

$$\begin{array}{c} E(K)=0.5\cdot l\cdot E(Z)+(1-E(H)) \end{array}$$ (10)

Eq (10) corresponds to half of the number of IBD block edges, i.e. half of the number of external junctions over *l* Morgans plus half of the number of chromosome tips for which a fraction 1-E(H) is IBD. Injecting Eqs (9) and (10) into our Eq (7), we obtain that for a chromosome of finite length *l*:

$$\begin{array}{c} E\left(L\right)=\frac{l\cdot (1-E(H))}{0.5\cdot l\cdot E(Z)+(1-E(H))} \end{array}$$ (11)

One may wish to use the moments, of *L* in statistical inferences from population data, and for instance develop a neutrality test. Let us consider a pseudo-dataset obtained from simulations of the same population model as in Stam [15]. When simulating *R* replicates, for one generation, it is possible to measure the mean length of IBD blocks in this dataset in three ways:

$$\begin{array}{ccc} L_{AR} & = & \frac{\sum_{r=1}^{R}\sum_{i=1}^{N}\sum_{j=1}^{k_{r,i}}l_{r,i,j}}{\sum_{r=1}^{R}\sum_{i=1}^{N}k_{r,i}}\\ L_{PW} & = & \frac{1}{R}\sum_{r=1}^{R}\frac{\sum_{i=1}^{N}\sum_{j=1}^{k_{r,i}}l_{r,i,j}}{\sum_{i=1}^{N}k_{r,i}}=\frac{1}{R}\sum_{r=1}^{R}L_{PW,r}\\ L_{IW} & = & \frac{1}{R}\sum_{r=1}^{R}\frac{1}{N}\sum_{i=1}^{N}\frac{\sum_{j=1}^{k_{r,i}}l_{r,i,j}}{k_{r,i}}=\frac{1}{R}\sum_{r=1}^{R}\frac{1}{N}\sum_{i=1}^{N}L_{IW,r,i} \end{array}$$

where *k*_*r*, *i*_ is the number of IBD blocks in the individual *i* of the replicate *r* and *l*_*r*, *i*, *j*_ is the length of the block *j* in the individual *i* of the replicate *r*. *L*_*AR*_ is a measure over all the replicates and therefore we have only one value for a whole dataset. *L*_*PW*_ is the mean over the replicates of *L*_*PW*, *r*_ that is a population-wise measure for which we have one value per population. *L*_*IW*_ is the mean over all the individuals in all the replicates of *L*_*IW*, *r*, *i*_ that is an individual-wise measure for which we have one value per individual and a whole distribution per population.

**Fig 2:**
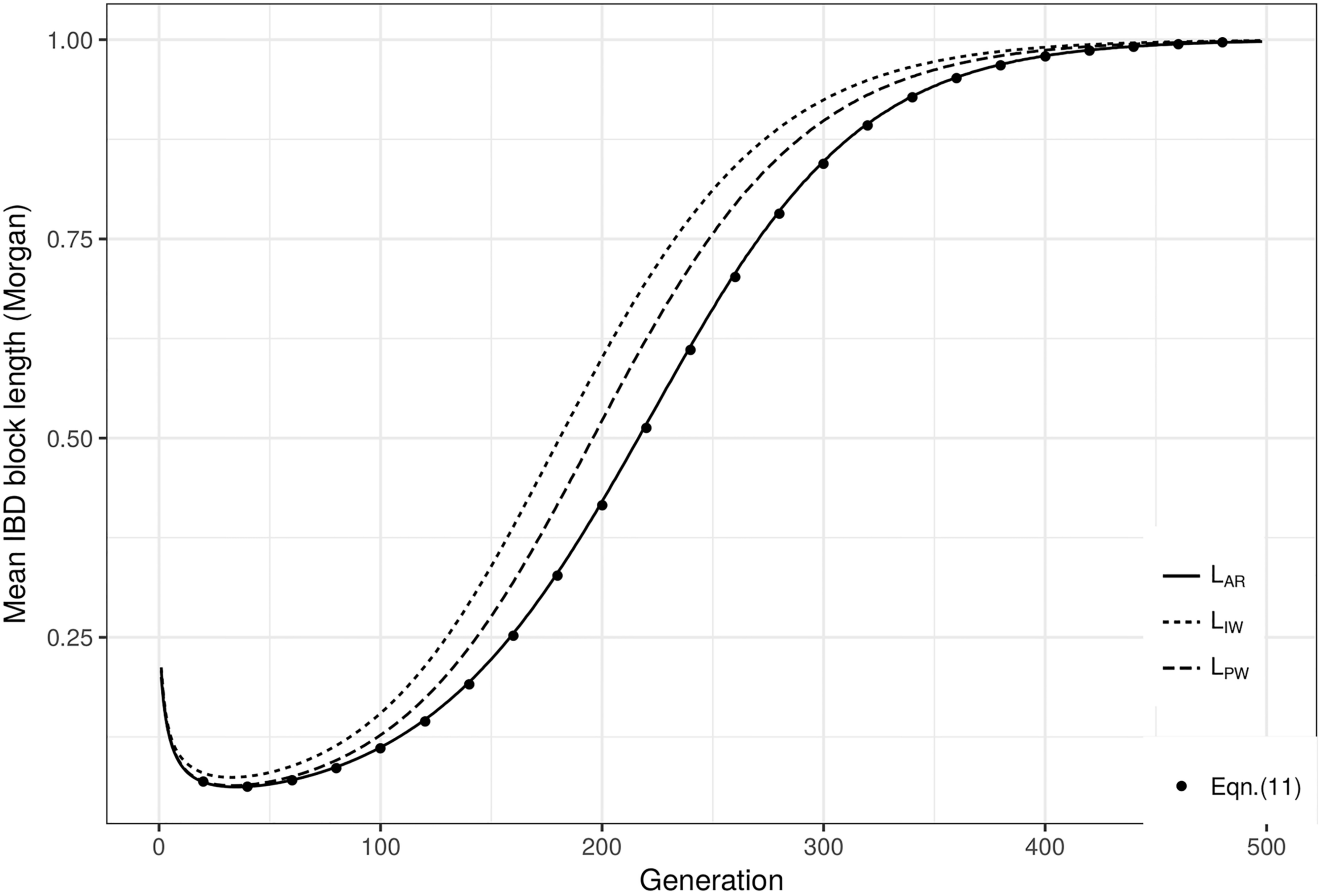
Comparing the different measures *L*_*AR*_, *L*_*PW*_ and *L*_*IW*_ in lines and the prediction of Eq (11) in dots. These values were obtained from simulations of a population of *N* = 20 diploid individuals, with a chromosome length of *l* = 1 Morgan, over 500 generations. 1,000,000 replicates were simulated.

On Fig 2 that shows all the different measures and prediction, we could see that *L*_*AR*_ is very close to E(L) of Eq (11), and it is indeed easy to prove mathematically why the former tends towards the latter when the number of replicates tends towards infinity. We have therefore developed a formula, E(L) of Eq (11), to very well predict *L*_*AR*_, as shown on Fig 2. We could also see that these measures are different, because they are indeed all the mean lengths of IBD blocks randomly drawn, but from different samplings: *L*_*AR*_ is the mean length of an IBD block drawn from the whole pseudo-dataset; *L*_*PW*_ is of a block drawn from a randomly drawn population of the dataset; and *L*_*IW*_ is of a block drawn from a randomly drawn individual of the dataset. Since the number of IBD blocks is different in each population and each individual, these samplings, and so these measures, are different and size-biased. Similarly, we could see that the asymptotic value of *L*_*AR*_, that is E(L) of Eq (11), is a lower bound of *L*_*PW*_ and *L*_*IW*_: we then have a theoretical formulation for what appears to be a lower bound of *L*_*PW*_ and *L*_*IW*_. This relation is yet to be mathematically proven.

## 3 Discussion

In this paper, we have reviewed two types of forward models commonly used to study theoretically the evolution of IBD blocks of chromosomes in a population, and have shown how these models are complementary. Models based on a random walk on a hypercube are very powerful to provide exact formula about the distribution of the total IBD length, but are only available for some very particular pedigrees. On the other hand, models based on a renewal process are very powerful to consider more general population models, but only means of IBD quantities have been obtained so far. We have provided a general formula for the mean IBD block length with Eq (7), that is independent of the demographic structure of the population or any evolutionary pressure. It is moreover the exact asymptotic value of *L*_*AR*_, and an asymptotic lower bound of *L*_*PW*_ and *L*_*IW*_.

When studying real data, one should be aware of the difference between the aforementioned measures (*L*_*AR*_, *L*_*PW*_ and *L*_*IW*_) before developing the appropriate statistical test. If IBD blocks are sampled from one or several populations without any constraint, the appropriate measure will be *L*_*AR*_ and the corresponding prediction E(L). If IBD blocks are sampled from one or several populations, but drawing the same number of IBD blocks from each population, the appropriate measure will be *L*_*PW*_. Finally, if IBD blocks are sampled from one or several populations, but drawing the same number of IBD blocks from each individual, the appropriate measure will be *L*_*IW*_. When there is no replicate in real data, there is no practical difference between *L*_*AR*_ and *L*_*PW*_. Their asymptotical distributions are not the same however, so that one could develop two different tests for the same measure, and pick the most appropriate one depending on the sampling policy. When there are replicates, *L*_*AR*_ and *L*_*PW*_ are indeed different. However, apart from the sampling policy, choosing between *L*_*AR*_ and *L*_*PW*_ is arbitrary. Further studies more thoroughly describing the distributions of *L*_*AR*_ and *L*_*PW*_ should help to make this choice no more arbitrary. Finally, exact theoretical formulations of *L*_*PW*_ and *L*_*IW*_ are yet to be discovered, and therefore, further work should also focus on completing this theoretical framework to make the study of any kind of real population datasets possible.
